# Supplementary material for: Impact of Face Mask-Wearing on Quality of Life in Post-Surgical Oral Cancer Patients: A Cross-Sectional Study
Source: Cancers (Basel). 2024 Dec 17;16(24):4199. doi: 10.3390/cancers16244199 (PMC11674196; doi:10.3390/cancers16244199)
Supplement: Supplementary file 1 [file cancers-16-04199-s001.zip › Supplementary File S2 questionnaire mask.pdf]

Influence of wearing a mask on the quality of life of patients with cancer of the upper aerodigestive tract

PATIENT NUMBER

	Mask conditions	Never	Seldom	Some of the time	Most of the time
**Q1 Appearance : Can you forget what you look like?**	With				
	Without				
**Q2 Fear of family's perception : Are you afraid of the way your friends and family look at you?**	With				
	Without				
**Q3 Fear of other's perception : Are you afraid of the way strangers look at you?**	With				
	Without				
**Q4 Fear of being photographed : Are you worried about being photographed?**	With				
	Without				
**Q5 Fear of others' judgment : Are you worried about the judgement of others ?**	With				
	Without				
**Q6 Fear of hurtful comments : Are you afraid that people will make hurtful comments?**	With				
	Without				
**Q7 Self-confidence : Do you lack self-confidence ?**	With				
	Without				
**Q8 Appearance-related depression: Are you depressed because of the way you look?**	With				
	Without				
**Q9 Sociability apprehension : Do you find it easier to talk and meet other people?**	With				
	Without				
