# Supplementary figures and images for: Impact of Face Mask-Wearing on Quality of Life in Post-Surgical Oral Cancer Patients: A Cross-Sectional Study
Source: Cancers (Basel). 2024 Dec 17;16(24):4199. doi: 10.3390/cancers16244199 (PMC11674196; doi:10.3390/cancers16244199)

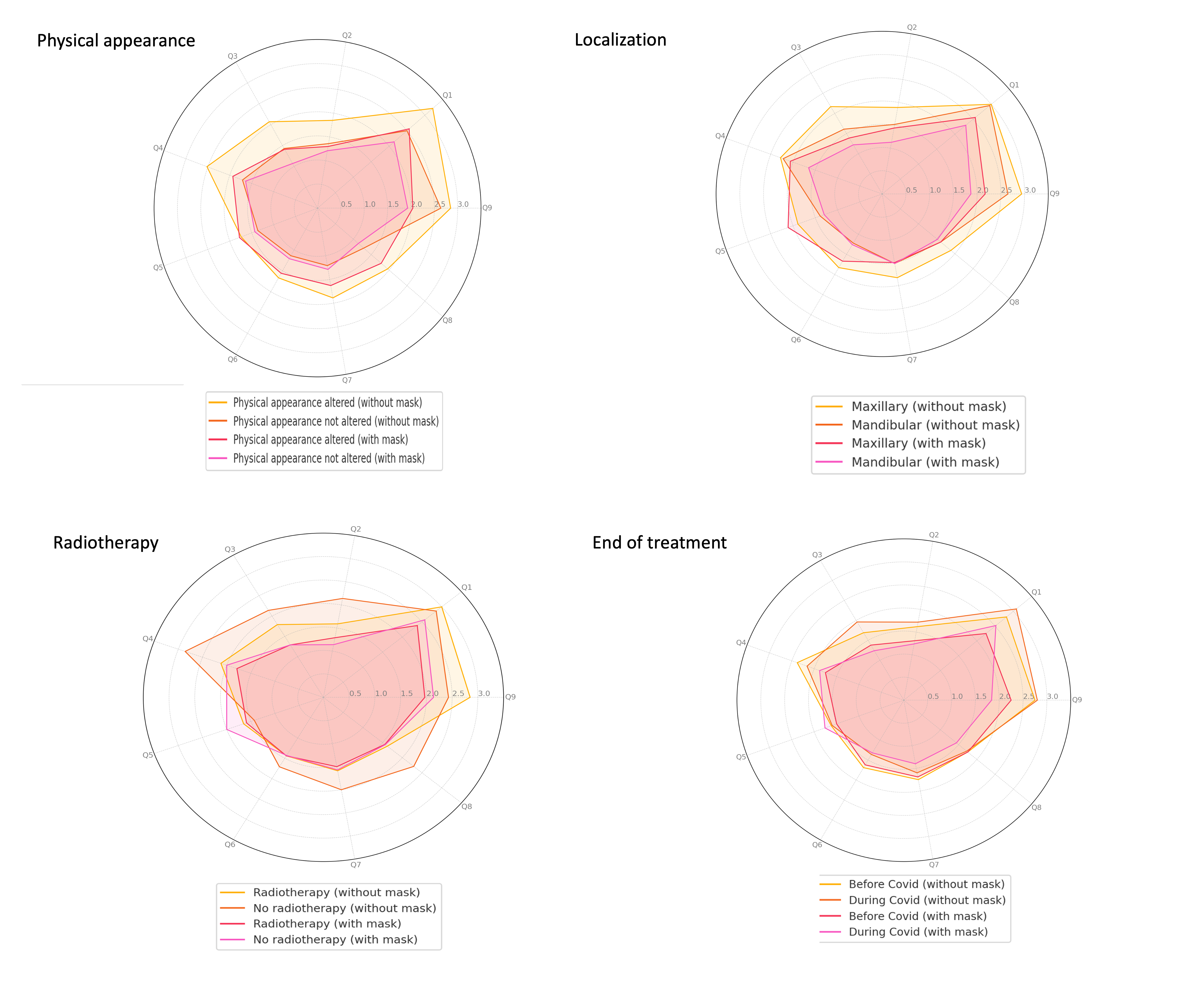

Supplement: Supplementary file 1 [file cancers-16-04199-s001.zip › Supplementary File S4 Spider plot.png]
